# Annual Meeting of the Fillmore County, Minn., Medical Society

**Published:** 1869-07-15

**Authors:** 


### *Annual Meeting of the Fillmore County, Minn., Medical Society.*

The Society met June 7th, at the office of Dr. Redman, in Preston, and was called to order by the President.

Dr. Redman moved to elect delegates to attend the State Medical Association, at Owatonna. Passed. Drs. R. W. Twitchell, A. H. Trow, L. Redman, L. Miller, and M. Donnelly were elected.

The following resolutions were then introduced and adopted:

*Resolved*, That the code of ethics adopted by the American Medical Association shall be our guide in our professional intercourse.

*Resolved*, That we will not fraternize with, nor meet in consultation, any one except a graduate of some regular medical college, who sustains a good moral character, provided this does not exclude a student reading with a member of this society, nor a physician who has been approbated by the Board of Censors of some medical society of the state of Minnesota.

*Resolved*, That each member of this Society shall inform the President thereof, in writing, at what college he graduated, giving the date of his diploma, on or before July 10th, 1869; and should any one neglect to comply with the provisions of this resolution, his name shall be stricken from the constitution; and, be it further

*Resolved*, That no person shall hereafter become a member of this Society until he has complied with the above requirement, or is admitted to membership by a vote of the Society.

*Resolved*, That Dr. Redman is hereby requested to prepare a list of the regular graduates of the county, also a list of the irregular practitioners and forward the same to Dr. Hand, of St. Paul.

WHEREAS, Medical societies are organized for diffusion of knowledge and mutual benefit of the members; therefore,

*Resolved*, That each member will be expected to read an essay on some medical subject, or report the history of some case, as occurred in his practice, for discussion at each meeting of the Society.

The following officers were elected for the ensuing year. R. W. Twitchell, President; Dr. L. Redman, Vice President; Dr. A. H. Trow, Treasurer; Dr. M. Donnelly, Secretary; Drs. L. Redman, L. Miller, and — Plummer, Board of Censors.

Dr. Redman moved that the Secretary forward copies of proceedings to the Chicago Medical Journal and the County papers for publication. Carried.

Motion to adjourn, to meet on the second Monday of July, 1870, carried.

